# A Portable Magnetoelectric Gaussmeter Based on Torque Effect

**DOI:** 10.3390/s25030855

**Published:** 2025-01-31

**Authors:** Jingen Wu, Jiacheng Qiao, Xianfeng Liang, Yongjun Du, Jieqiang Gao, Yiwei Xu, Jinghong Guo, Min Lu, Ming Zhang, Zhongqiang Hu

**Affiliations:** 1 State Key Laboratory for Manufacturing Systems Engineering, Electronic Materials Research Laboratory, Key Laboratory of the Ministry of Education, Engineering Research Center of Spin Quantum Sensor Chips, Universities of Shaanxi Province, School of Electronic Science and Engineering, Xi’an Jiaotong University, Xi’an 710049, Chinazhongqianghu@xjtu.edu.cn (Z.H.); 2 Institute of Electric Power Sensing Technology, China Electric Power Research Institute Co., Ltd., Beijing 100192, China; 3 State Grid Jiangsu Electric Power Co., Ltd., Nanjing Power Supply Company, Nanjing 210000, China

**Keywords:** portable device, magnetoelectric gaussmeter, torque effect

## Abstract

A giant magnetoelectric coefficient has been discovered in laminated magnetoelectric composites incorporating piezoelectric and magnetostrictive layers, which reveals a high sensitivity in AC magnetic field detection under a DC bias field. However, the DC-biased magnetoelectric composites are not capable of detecting DC magnetic fields due to the interference with the DC signal to be measured. Here, we demonstrate a portable magnetoelectric gaussmeter based on torque effect that can detect both DC and AC magnetic fields. The proposed gaussmeter is equipped with a magnetoelectric sensor, a charge amplification module, a signal processing circuit, a power module, a data processing program, a display module, etc. The proposed gaussmeter indicates such performance indexes as an intensity range of 0~10 Oe, frequency range of DC~500 Hz, AC detection limit of 0.01 Oe, DC detection limit of 0.08 Oe, and frequency resolution of 1 Hz. Being powered by a power adapter (or a battery) of 5V 2A, the whole device system is pocket-size, low-cost, and highly portable, demonstrating its potential for magnetic field detection as a distributed sensor.

## 1. Introduction

Magnetoelectric (ME) effect refers to such a phenomenon where varied magnetization induces electric polarization, or, vice versa, where varied electric polarization induces magnetization [1,2,3,4,5,6]. Taking advantage of the coupling between electric and magnetic fields, the device based on magnetoelectric effect can further evolve into electric and/or magnetic field sensors. Through the further development of these magnetoelectric sensors, various device applications, such as a current sensor, electronic compass, gyrator, isolator, energy harvester, magnetoelectric antenna, etc., have been realized [7,8,9,10,11]. Compared with other magnetic field sensors, including a Hall sensor, magnetoresistive23 sensor, magneto-diode, search coil, etc., refs. [12,13,14,15,16,17,18] the magnetoelectric-type magnetic field sensors reveal evident advantages, such as high sensitivity, high output, and high energy conversion efficiency [19,20,21,22]. Thus, various magnetoelectric devices that can be used to detect magnetic fields have been proposed. Zhai et al. designed a flexible magnetoelectric Metglas/PVDF (polyvinylidene fluoride) composite, with a DC magnetic field sensitivity reaching up to 2 nT [23]. Resi et al. developed a DC magnetic field sensor based on the magnetoelectric (ME) PVDF/Metglas composite, which revealed a linear response in a 0 Oe to 2 Oe DC magnetic field range, with a 70 nT resolution [24]. Yang et al. demonstrated an arc-shaped Metglas/polyvinylidene fluoride (PVDF)/Ni laminate, which exhibited a large self-biased magnetoelectric (ME) effect, with a limit of detection (LOD) of up to 8 nT at 1 Hz [25].

In addition, MEMS-type magnetoelectric sensors based on AlN piezoelectric films have been extensively studied. Greve et al. fabricated a thin film magnetoelectric composite, which consists of aluminum nitride AlN and (Fe_90_Co_10_)_78_Si_12_B_10_ ferromagnetic metallic alloy, using magnetron sputter deposition. The fabricated thin film magnetoelectric composite revealed ME coefficients of 737 V/cm Oe at mechanical resonance and 3.1 V/cm Oe out of resonance [26]. Yarar et al. investigated the magnetoelectric thin film sensors made of FeCoSiB/AlN heterostructures, which revealed a giant ME coefficient as high as 5 kV/cm Oe with a low limit of detection reaching 400 fT/Hz^1/2^ at mechanical resonance [27] Li et al. reported an NEMS DC/low-frequency magnetic field sensor consisting of a AlN/FeGaB resonator, which presented a high DC magnetic field sensitivity of 2.8 Hz/nT and a limit of detection of 800 pT [28]. Hayes et al. demonstrated converse magnetoelectric modulation in the piezoelectric AlN and magnetostrictive FeCoSiB composite, which revealed an AC field sensitivity up to 64 kV/T, with a DC limit of detection of 210 pT/Hz^1/2^ and an AC limit of detection of 70 pT/Hz^1/2^ at 10 Hz [29].

The excellent performances of these magnetoelectric sensors are fundamentally dominated by the magnetoelectric coupling properties. Thus, the magnetoelectric sensors can be further promoted in their performance via enhanced magnetoelectric coupling. Commonly, the magnetoelectric coupling effect can be enhanced by ways such as optimizing material performance, innovating process route, introducing new coupling mechanisms, refining device structure, etc. Chu et al. demonstrated a (1-1) connected magnetoelectric composite made of piezoelectric/magnetostrictive fibers which were annealed by laser, and the composite revealed an extremely high AC field sensitivity of 0.135 pT at ~23.23 kHz [30]. Dong et al. proposed a magnetoelectric composite consisting of the PZT/interdigital electrode (IDE) core with highly magnetostrictive Metglas foils, and the laminated composite was compacted in a vacuum bag and cured for 12 h at room temperature. Their proposed sensor revealed an equivalent magnetic noise as low as 260 fT/Hz^1/2^ at 23.95 kHz [31]. Wang et al. designed an ME sensor consisting of a six-layered magnetostrictive Metglas and a piezoelectric transducer, where the Metglas layers were symmetrically stacked and bonded to both the top and bottom sides of the interdigital electrode/PMN-PT fiber with epoxy resin, using a vacuum bag pressure method. With the reduction in internal sensor noise sources, an extremely low equivalent magnetic noise of 5.1 pT/Hz^1/2^ was found at 1 Hz [32].

Even though the reported magnetoelectric composites have presented great potential for magnetic field detection, there are still many obstacles in the course of practical application for these magnetoelectric composites. For instance, the laminated magnetoelectric composites make use of magnetostrictive materials, such as Metglas, Terfenol-D, rare earth alloy compounds, etc. These magnetostrictive materials always need a DC bias magnetic field so that they are sensitive to the AC magnetic field. However, the DC bias magnetic field is always provided by a bias coil or permanent magnets, which causes high power consumption and bulk volume. Also, the DC bias magnetic field induces the interference with the DC signal. To remove the DC bias, self-biased structures in magnetoelectric composites have been developed. As is widely known, the self-biased property can be obtained by combining magnetostrictive materials with different coercive fields [33]. Nan et al. demonstrated a magnetoelectric nano-electromechanical system (NEMS) resonator based on the AlN/(FeGaB/Al_2_O_3_) magnetoelectric heterostructure, which led to a self-biased detection mechanism with a low limit of detection of DC magnetic fields of 300 pT [34]. Li et al. reported that the self-biased Metglas/Pb(Zr,Ti)O_3_/Metglas laminates by inducing remanent magnetization in annealed Metglas to generate an internal bias field, where the ME coefficients under 1 kHz and electromechanical resonance are 12 V/cm Oe and 380 V/cm Oe, respectively [35]. Zhang et al. developed the trilayer Terfenol-D/PZT-5H/SmFe_2_ magnetoelectric heterostructure operating in shear mode, which revealed a high non-zero ME voltage coefficient of 2.24 V/Oe due to the large intrinsic anisotropic field of SmFe_2_ plate [36]. Wu et al. proposed a self-biased magnetoelectric sensor operating in d_36_ face-shear mode, in which the self-biased effect was induced by the residual stress at the interface of magnetostrictive and piezoelectric materials. The d_36_ face-shear mode magnetoelectric sensor revealed a high ME coefficient of 48.8 V/cm Oe at resonant frequency under zero DC bias [37].

Without doubt, such magnetostrictive composites reveal extremely high AC sensitivities. While in the situation of DC magnetic detection, the applied DC bias magnetic field induces crosstalk with the DC signal to be measured. Also, the DC bias magnetic field is in an optimized range; when the DC magnetic field under test exceeds such a range, the DC sensitivity is seriously suppressed. Apart from these magnetoelectric composites working under a DC bias field, the magnetoelectric composites that operate in torque effect do not need a DC bias field and present sensitivities to both DC and AC magnetic fields, which also have great potential for magnetic field detection [38]. With high energy conversion efficiency, the torque effect has also been utilized to design energy harvesters. Peddigari et al. designed a magneto-mechano-electric energy harvester based on torque effect, with a high output power of 7.4 mW and an equivalent power density of 0.58 mW/cm^3^ Oe under a 3 Oe magnetic field [39]. Kwak et al. proposed a vertically installed magneto-mechano-electric energy harvester operating in torque effect, which generated a 12.2 mW output power from a low-amplitude stray magnetic field of 2.5 Oe [40]. Yu et al. developed an X-shaped magneto-mechano-electric energy harvester utilizing a bionic fourfold symmetric structure, which achieved a record high output power of 7.31 mWRMS under a 0.75 Oe magnetic field [41]. Kim et al. proposed a hybrid magneto-mechano-electric energy harvester combining the magnetic torque effect and electromagnetic induction effect, where the output power was 28.35 mW with a power density of 0.042 mW/cm^3^ Oe^2^ in a 5 Oe magnetic field of 60 Hz [42].

In this work, we develop a portable gaussmeter based on torque effect. The proposed device is integrated with a magnetoelectric sensor, a charge amplification module, a signal processing circuit, a power module, a data processing program, etc. Based on the processing program that burned into the single chip microcomputer (STM32F103 Core Board), the proposed gaussmeter can realize such functions as reading, storage, analysis, processing, display, etc. The proposed portable gaussmeter is theoretically and experimentally investigated, proving its potential for magnetic field detection in distributed application environments.

## 2. Structure Design and Theoretical Analysis

### 2.1. Structure Design

**Figure 1:**
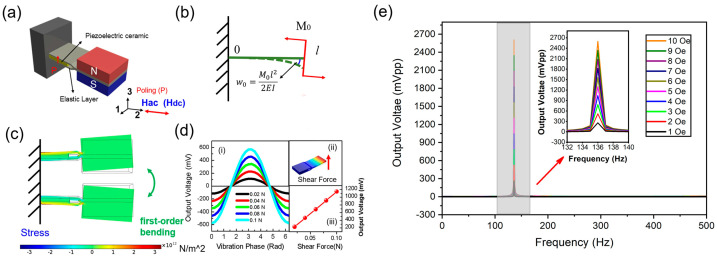
(**a**) Schematic diagram of the sensor that is used in our proposed gaussmeter. (**b**) Mechanical analysis of the sensor operating in torque effect. (**c**) The finite element simulation of the stress distribution under first-order bending mode. (**d**) The finite element simulation of output voltage with respect to the shear force: (**i**) the waveform of output voltage under different shear force, (**ii**) the schematic diagram of shear force, (**iii**) the peak-to-peak value of output voltage as a function of shear force. (**e**) The finite element simulation of output voltage with respect to frequency, under different applied AC magnetic fields.

Figure 1a presents the schematic diagram of the sensor that is used in our proposed gaussmeter. As can be seen, the proposed sensor consists of an elastic layer, a piezoelectric layer, and a pair of NdFeB permanent magnets. The piezoelectric layer is made of PZT-5H piezoelectric ceramic with dimensions of 5 mm (length) × 4 mm (width) × 0.3 mm (thickness). After its top and bottom surfaces are electroded by annealed silver paste, the piezoelectric layer is poled along the thickness direction, which is denoted as direction 3. PZT-5H piezoelectric ceramic is sandwiched by two elastic layers. After that, one end of the laminated structure is clamped, which functions as the fixed end of a cantilever beam. A pair of NdFeB permanent magnets is bonded at the other end of the laminated structure. The piezoelectric layer, elastic layers, and permanent magnets are bonded together by epoxy resin, which is cured at room temperature for 24 h. The magnet’s magnetization direction is along its thickness direction, which is also denoted as direction 3. The whole structure is sensitive to the transversal AC and DC magnetic fields (along direction 2), which is ascribed to the magnetic torque.

The mechanical analysis of the cantilever is presented in Figure 1b. Under a transversal magnetic field, a magnetic torque *M*_0_ is induced at the free end. Then, a shear force is subjected to the free end, leading to a small deflection (i.e., *w*_0_). The magnetic torque *M*_0_ and the small deflection *w*_0_ is estimated via the following equations [43,44]:

(1) $$M_{0}=m\times B\approx \left(B_{r}\cdot V\right)\times B,$$

(2) $$w_{0}\left(x\right)=\frac{M_{0}x^{2}}{2\bar{E}I},\;x\in \left[0,l\right],$$

where ***B*** is the transversally applied magnetic field, ***m*** is the magnetic moment of permanent magnets, *V* is the total volume of magnets, and ***B_r_*** is the residual flux density of the magnet. $w_{0}\left(x\right)$ is the cantilever deflection, where *x* refers to the distance away from the fixed end, *x*∈[0, *l*]. *l* is the length of the cantilever. $\bar{E}=\frac{E_{m}E_{e}}{V_{m}E_{e}+V_{e}E_{m}}$ is the equivalent elasticity modulus, where *E*_m_ is the modulus of the elastic layer and *E*_e_ is the modulus of epoxy. *V*_m_ is the volume fraction of the elastic layer, and *V*_e_ is the volume fraction of epoxy [45]. $I=\frac{wh^{3}}{12}$ is the total inertia moment, where *w* and *h* are the total width and total thickness of the cantilever, respectively.

As for the cantilever with one end clamped, its resonant frequency *f*_n_ for different bending modes is estimated by [46]

(3) $$f_{n}=\frac{\pi h}{4\surd 3l^{2}}\cdot \sqrt{\frac{1}{\bar{\rho }\bar{s_{22}}}}{\cdot \beta }_{n}^{2},$$

where *h* is the total thickness of the cantilever, *l* is the length of the cantilever, and $\bar{\rho }=V_{e}\rho_{e}+V_{m}\rho_{m}$ is the average density of the cantilever. $\rho_{m}$ and $\rho_{e}$ are, respectively, the densities of the elastic layer and epoxy. $\bar{s_{22}}=1/\bar{E}$ is the equivalent transverse modulus of the composite. $\beta_{1}=0.597,\;\beta_{2}=1.494,\cdots \cdots ,\beta_{n}\approx n-0.5,$ where *n* is the order of the bending mode.

### 2.2. Finite Element Simulation

When the free end of the cantilever periodically vibrates, a shear force will be induced at the fixed end, which is confirmed by the finite element simulation (COMSOL Multiphysics, COMSOL, Inc., Stockholm, Sweden). The material parameter that is used for the finite element simulation is provided by the material manufacturer or the COMSOL material database, as listed in Table 1. As shown in Figure 1c, when the cantilever vibrates under the first-order bending mode, stress will be induced in the elastic layer. Such stress originates from the vibration of the tip mass; when it transmits to the fixed end, the shear force that is applied to the piezoelectric layer is generated. The shear force is calculated as [38]

(4) $$F_{S,x=0}=\frac{M_{0}\omega^{2}l^{2}m}{6\bar{E}I},$$

where $F_{S,\;x=0}$ is the shear force at the fixed end, $\omega =2\pi f_{n}$ is the angular velocity of vibration, and *m* is the total mass of the cantilever, taking into account the mass of permanent magnets.

According to the piezoelectric effect, we have the following equations:

(5) $$Q_{3}={d_{33}\cdot F}_{S,x=0},$$

(6) $$V_{3}=\frac{Q_{3}}{C_{0}}=\frac{{d_{33}\cdot F}_{S,x=0}}{C_{0}},$$

where $d_{33}$ is the piezoelectric coefficient and $V_{3}$ is the induced voltage on the piezoelectric layer, which is induced by the magnetoelectric (ME) coupling effect and thus is named as ME voltage. $Q_{3}$ is the total charge on the piezoelectric layer, and $C_{0}$ is the capacitance of the piezoelectric layer. It can be directly deduced that the ME voltage is in direct proportion to the shear force, according to Equation (6). This relation is also confirmed by the finite element simulation, as shown in Figure 1d. The simulated waveforms of ME voltage under different shear forces, i.e., 0.02 N, 0.04 N, 0.06 N, 0.08 N, 0.1 N, are presented in Figure 1(d-i). It is found that the ME voltage increases with the increased shear force. Figure 1(d-ii) shows the schematic diagram of shear force generated by a load at the cantilever free end. Figure 1(d-iii) demonstrates the simulated relation between the peak-to-peak value of the ME voltage and the amplitude of the shear force. The simulated result is in accordance with Equation (6), where the ME voltage is linearly proportional to the shear force. Furthermore, the finite element simulation of output voltage with respect to frequency, under different applied AC magnetic fields, i.e., magnetic torque $M_{0}$, is presented in Figure 1e. As for the shear force $F_{S,x=0}$, this is induced by the magnetic torque $M_{0}$. Different magnetic torques $M_{0}$ will induce different shear forces, and the shear force is in direct proportion to the magnetic torque. According to Equation (3), the resonance frequency is determined by the material and structure parameter, rather than the shear force. Thus, the magnetic torque $M_{0}$ (or shear force) has little effect on the resonance frequency; this is proven by the finite element simulation.

By substituting Equations (4) and (5) into Equation (6), we can obtain the relation between $V_{3}$ and the magnetic torque *M*_0_ as follows:

(7) $$V_{3}=\frac{M_{0}\omega^{2}l^{2}md_{33}}{6\bar{E}IC_{0}},$$

where $d_{33}$ and $C_{0}$ are constants for the piezoelectric materials at room temperature. As for a certain frequency, e.g., the resonant frequency, ω is a fixed value. Thus, the ME voltage $V_{3}$ is also in direct proportion to the magnetic torque *M*_0_. Bear in mind that the magnetic torque *M*_0_ has such a relation with the external magnetic field, i.e., $M_{0}=m\times B\approx \left(B_{r}\cdot V\right)\times B$, where $B_{r}$ and *V* are constants. Then, the magnetic torque *M*_0_ is merely decided by the intensity of the external magnetic field. Accordingly, it is further deduced that the ME voltage can be used to measure the intensity of the external magnetic field.

## 3. Results and Discussion

### 3.1. Frequency Dependence

**Figure 2:**
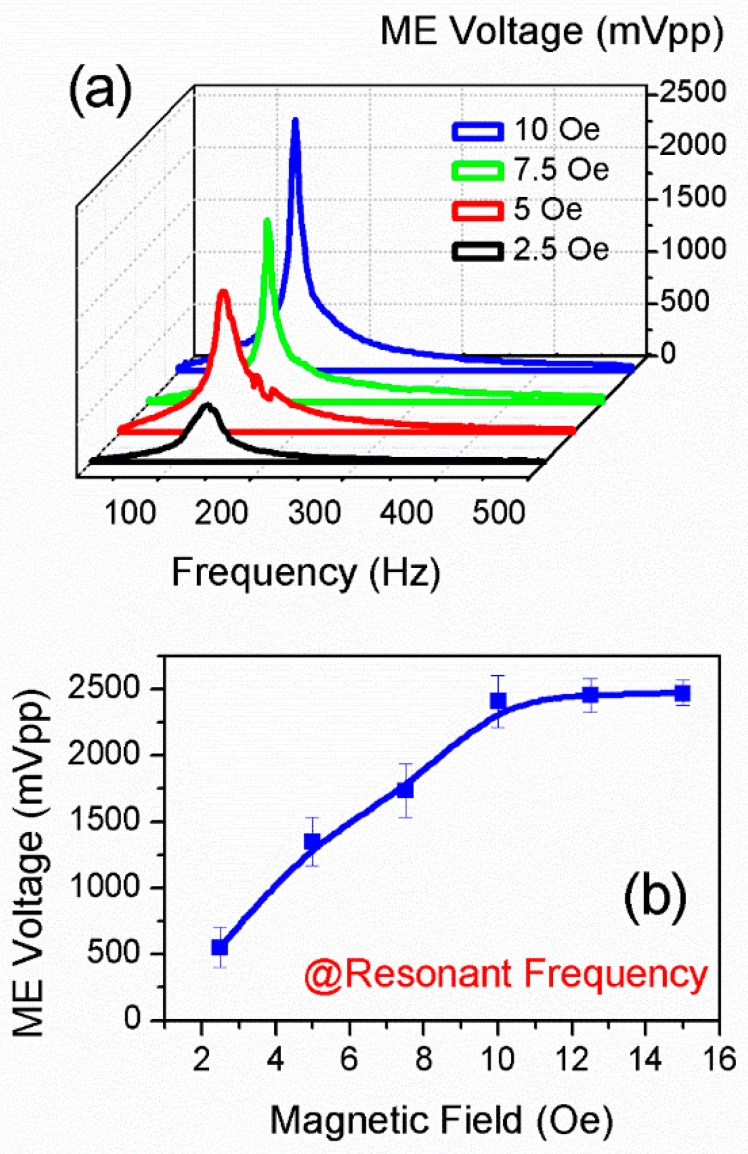
(**a**) The variation in ME voltage as a function of frequency under AC magnetic fields of 2.5 Oe, 5 Oe, 7.5 Oe, and 10 Oe within the frequency range of 10 Hz to 500 Hz. (**b**) The variation in ME voltage with respect to magnetic field intensity under resonant frequency.

The variations in the ME voltage as a function of frequency are shown in Figure 2a and are within the frequency range of 10 Hz to 500 Hz. As can be seen, four curves of ME voltage are indicated, where the RMS (Root Mean Square) values of the magnetic field intensity for those four curves are 2.5 Oe, 5 Oe, 7.5 Oe, and 10 Oe, respectively. The ME voltage reveals a peak shape variation in the test frequency range, where the maximum ME voltage appears around the resonant frequency, corresponding to the first-order bending mode of the sensor. The resonant frequency of the first-order bending mode is around 134.95 Hz. Although there exist small fluctuations, the resonant frequencies under 2.5 Oe, 5 Oe, 7.5 Oe, and 10 Oe are comparatively close to 134.95 Hz, demonstrating that the first-order bending mode of the sensor is relatively stable under magnetic fields of different intensities.

Figure 2b shows the ME voltage under resonant frequency, where the magnetic field intensity gradually increases from 2.5 Oe to 15 Oe by intervals of 2.5 Oe. It can be seen that the ME voltage under resonant frequency reveals a linear increase when the magnetic field is below 10 Oe. Such linear variation in ME voltage is in well accordance with the theoretical derivation based on Equation (7), as well as the finite element simulation in Figure 1(d-iii). It should also be noted that when the external magnetic field exceeds 10 Oe, the measured ME voltage gradually reaches up to its saturation value. This can be ascribed to the fact that the laminated composite working under torque effect has a limit, around which the magneto-mechano-electric coupling efficiency cannot be further improved. Nevertheless, it still can be deduced that the proposed sensor can be used to measure AC magnetic fields below 10 Oe.

### 3.2. AC Sensitivity

**Figure 3:**
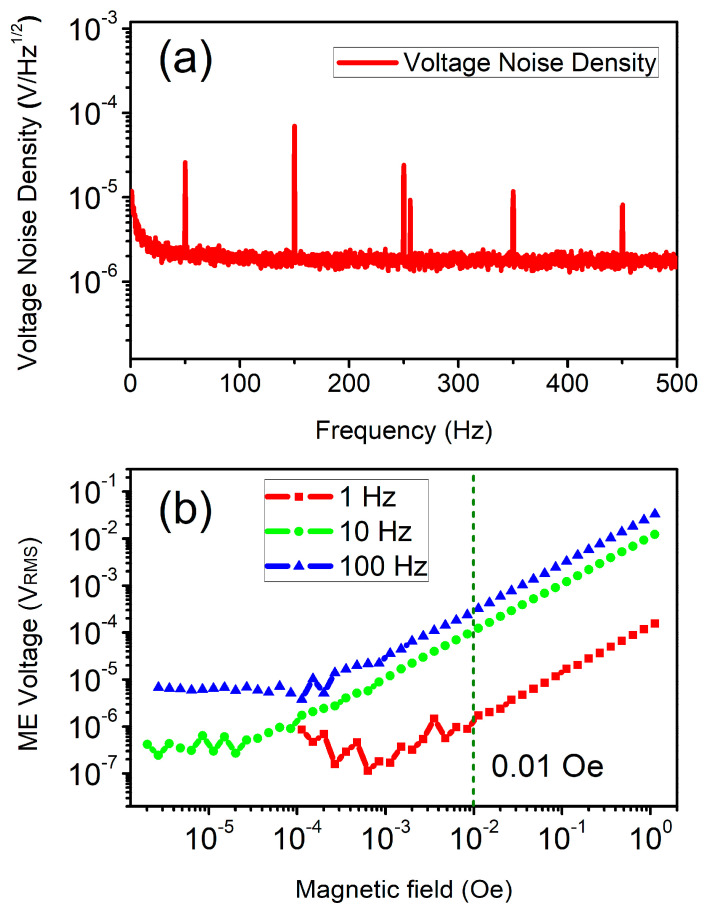
(**a**) Voltage noise density of the proposed sensor in the frequency range of 1 Hz to 500 Hz. (**b**) LOD (limit of detection) results for AC magnetic fields of 1 Hz, 10 Hz, and 100 Hz.

To indicate that the proposed sensor is capable of measuring the AC magnetic field, the voltage noise density and ME voltage are tested by a lock-in amplifier (SR865A, Stanford Research Systems, Sunnyvale, CA, USA), with a time constant of l second and a slope of 24 dB/oct, corresponding to an equivalent noise bandwidth (ENBW) of 78.125 mHz. The proposed sensor is placed in the shielding box during the voltage noise density test, with residual magnetization less than 1 nT and magnetic field disturbance less than 300 fT. The voltage noise density is acquired by normalizing the ME voltage to the square root of the ENBW. As can be seen in Figure 3a, the proposed sensor reveals a voltage noise density around 10^−5^ to 10^−6^ V/Hz^1/2^ in the frequency range of 1 Hz to 500 Hz. Also, the voltage noise density increases with the decreased frequency, indicating that the low-frequency noise is unavoidable. It should also be noted that the power frequency noise, as well as its odd harmonic noise, is non-negligible, which should be filtered through the signal processing method.

The AC sensitivity test of the proposed sensor is carried out, as revealed in Figure 3b. It is confirmed that the ME voltage is in direct proportion to the AC magnetic field intensity under different frequencies, i.e., the ME voltage reveals a linear variation with respect to the AC magnetic field of appropriate intensity. It is noted that the ME voltage under 10 Hz and 100 Hz still presents a linear variation when the AC magnetic field is as low as 0.001 Oe. In other words, the proposed sensor is able to detect AC magnetic fields of 10 Hz and 100 Hz, with a LOD below 0.001 Oe. Due to the existence of low-frequency noise, as shown in Figure 3a, the low-frequency performance of the proposed sensor is suppressed. The ME voltage under 1 Hz is not comparable to that under 10 Hz and 100 Hz. Nevertheless, the proposed sensor presents an LOD below 0.01 Oe under 1 Hz, which is still competitive with some Hall sensors [24,38].

**Figure 4:**
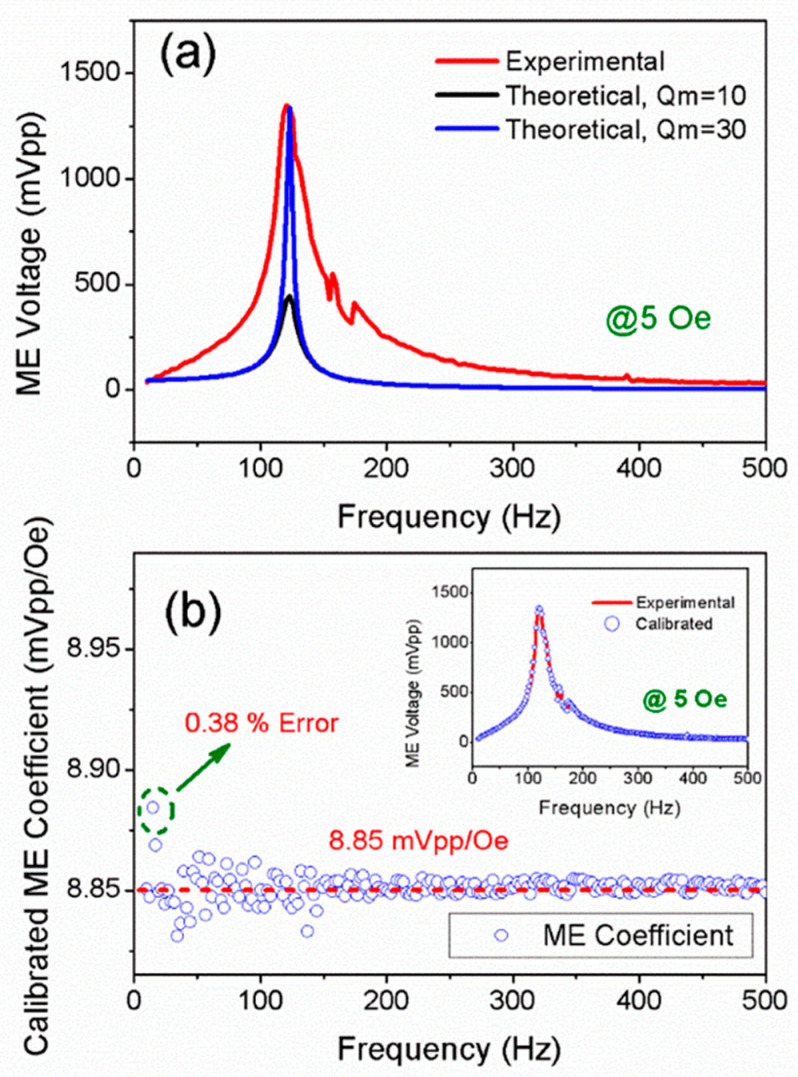
(**a**) The comparison between experimental and theoretical ME voltages under 5 Oe. (**b**) The calibrated ME coefficient is under 5 Oe; the inset is the calibrated ME voltage.

In addition, it should be noted that the ME voltage has a strong frequency dependence, i.e., it reveals a peak shape variation in the test frequency range, as shown in Figure 4a. Such frequency dependence originates from the first-order bending mode of the cantilever beam. When the cantilever beam vibrates at resonant frequency, the tip deflection will be amplified to *Q_m_* times, where *Q_m_* is the mechanical quality factor. Under such conditions, the tip deflection ratio of resonance versus off-resonance (i.e., $\frac{w_{r}}{w_{0}}$) can be expressed as [47]

(8) $$\frac{w_{r}}{w_{0}}=\frac{Q_{m}}{\sqrt{z^{2}+{\left(z^{2}-1\right)}^{2}Q_{m}^{2}}},$$

where *z* is the ratio of *f*_0_/*f*_r_, $w_{r}$ and $w_{0}$ are, respectively, the tip deflections of resonance and off-resonance, and *f*_r_ and *f*_0_ are, respectively, the resonant frequency and the off-resonant frequency. According to Equations (1), (2) and (7), it can be found that the ME voltage *V*_3_ is in direct proportion to both the magnetic torque *M*_0_ and tip deflection $w_{0}$(*x*). Then, we can obtain the frequency dependence of ME voltage as follows:

(9) $$V_{3}=\frac{1}{\sqrt{z^{2}+{\left(z^{2}-1\right)}^{2}Q_{m}^{2}}}\cdot \frac{M_{0}\omega^{2}l^{2}md_{33}}{6\bar{E}IC_{0}},$$

Based on Equation (9), the theoretical ME voltage as a function of frequency is given. *Q_m_* is a constant that determines the shape of the frequency spectrum. The comparison between the experimental and theoretical ME voltage is shown in Figure 4a, where the intensity of the external magnetic field is 5 Oe. By setting *Q_m_* as constants of 10 and 30, two frequency spectrums are obtained. It can be seen that theoretical ME voltages are not perfectly consistent with the experimental ones. This can be ascribed to the fact that the cantilever beam is not an ideal rigid body because it is made of different materials that are bonded together. Thus, the frequency dependence of ME voltage does not strictly obey the relation that was revealed by Equation (9). A modification must be introduced to make it more appropriate for the actual frequency dependence. Here, we propose a calibration method, where *Q_m_* is set as an array in the test frequency range, rather than a fixed constant. The calibrated *Q_m_* consists of various elements, i.e., [$Q_{m,1}$, $Q_{m,2}$, $Q_{m,3}$,……, $Q_{m,n}$], where $Q_{m,n}$ is the calibrated value for each testing frequency. $Q_{m,n}$ is acquired by the numerical iterative of Equation (9), where the theoretical ME voltage is calibrated with the experimental ME voltage. When the value difference between the theoretical and experimental ME voltages is within a certain margin of error, the value of $Q_{m,n}$ is determined. Based on the calibrated $Q_{m,n}$, the calibrated ME voltage $V_{3,cal}$ is calculated based on Equation (9).

Figure 4b presents the calibrated ME coefficient under 5 Oe. We use the following equation to calibrate the ME coefficient, i.e., $\alpha_{ME,\;cal}$.

(10) $$\alpha_{ME,\;\;cal}=\alpha_{ME,0}\cdot \frac{V_{3,exp}}{V_{3,cal}},$$

where $V_{3,exp}$ is the experimental ME voltage, $V_{3,cal}$ is the calibrated ME voltage, and $\alpha_{ME,0}$ is the average value of ME coefficients under off-resonant frequency. The inset of Figure 4b presents the calibrated ME voltage $V_{3,cal}$, which coincides well with the experimental value $V_{3,exp}$.

As can be seen in Figure 4b, the calibrated ME coefficient $\alpha_{ME,\;cal}$ is almost a fixed value within the test frequency range. Thus, it can be deduced that the frequency dependence of the ME coefficient can be eliminated by the calibration, which is beneficial to the intensity measurement of the magnetic field over a wide frequency range. The calibrated ME coefficient $\alpha_{ME,\;cal}$ is around 8.85 mVpp/Oe. Then, the external AC magnetic field intensity can be acquired via such an ME coefficient. It is worth mentioning that the testing error can be controlled at a level below 0.38%, demonstrating that the proposed calibration method is effective and precise enough to measure the intensity of the AC magnetic field.

### 3.3. DC Sensitivity

**Figure 5:**
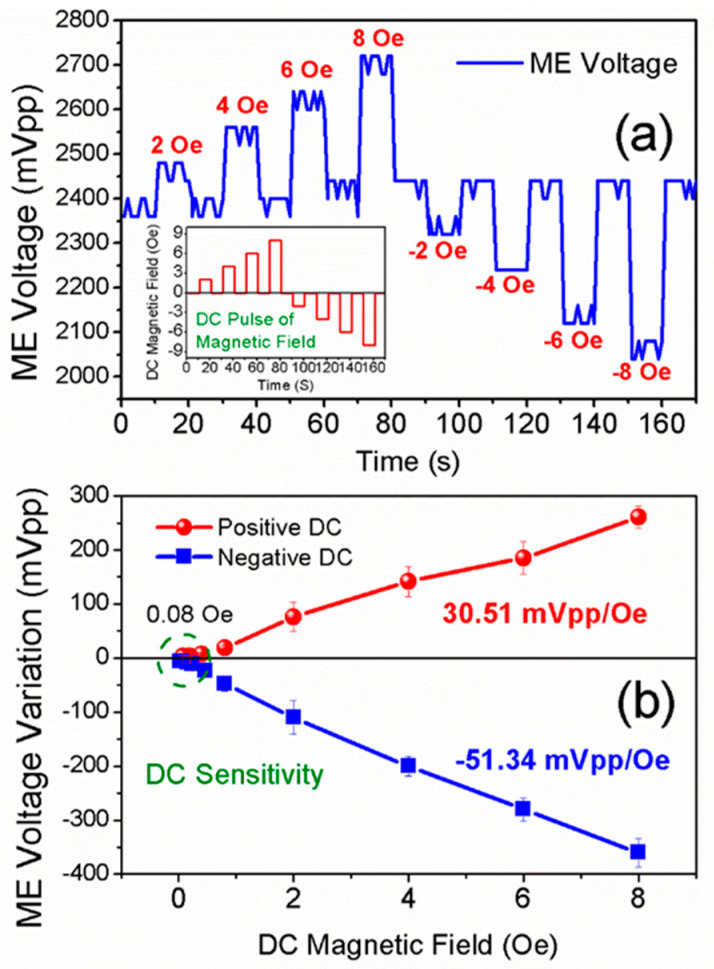
(**a**) The fluctuation of ME voltage under DC pulses of magnetic fields, where the intensities of the DC magnetic field are 2 Oe, 4 Oe, 6 Oe, 8 Oe, −2 Oe, −4 Oe, −6 Oe, and −8 Oe. (**b**) The variation in ME voltage as a function of both positive and negative DC magnetic fields.

Apart from AC magnetic field detection, the proposed sensor can also be used to detect DC magnetic fields. As shown in Figure 5a, the value of ME voltage reveals fluctuations under DC pulses of the magnetic field, where the proposed sensor is excited and vibrates under its first-bending mode via a 10 Oe AC bias magnetic field. The inset of Figure 5a presents the DC pulse of the magnetic field. As can be seen, the waveform of such a DC pulse is clearly reproduced by the ME voltage, indicating that the proposed sensor can detect DC magnetic fields. Compared with its initial value, the value of ME voltage reveals an increase under a positive DC magnetic field, in contrast to a decrease under a negative DC magnetic field. Meanwhile, it is also obvious that a DC magnetic field of higher intensity induces larger fluctuations in ME voltage. The ME voltage fluctuation that was induced by the ±8 Oe DC magnetic field is more prominent than those induced by ±6 Oe, ±4 Oe, and ±2 Oe DC magnetic fields. This can be ascribed to the ΔE effect in the magnetoelectric composite [48,49,50,51,52]. According to Equation (7), the ME voltage can be expressed in the following form:

(11) $$V_{3}=\frac{1}{\bar{E}}\frac{M_{0}\omega^{2}l^{2}md_{33}}{6IC_{0}},$$

where $\bar{E}$ is the equivalent elastic modulus of the cantilever beam. ω is the angular velocity frequency, which is a fixed value when the frequency of the AC bias magnetic field is unchanged. Expect for $\bar{E}$, other parameters in Equation (11) are constants when the piezoelectric materials, permanent magnet, device size, and AC bias magnetic field intensity are fixed. Under an external DC magnetic field, the equivalent elastic modulus $\bar{E}$ changes. As a result, the ME voltage changes. Such an effect is known as the ΔE effect, which is widely used in magnetoelectric composites for DC magnetic field detection [34,53,54,55].

As shown in Figure 5b, the ME voltage reveals a linear variation with respect to the external DC magnetic field. The ME voltage increases linearly under a positive DC magnetic field, while it decreases linearly under a negative DC magnetic field. And the DC sensitivities for both positive and negative DC magnetic fields can be estimated by the slope of ME voltage variation versus the DC magnetic field, i.e., ∆$V_{3}/∆H_{DC}$. The DC sensitivities for positive and negative DC magnetic fields are calculated to be 30.51 mVpp/Oe and −51.34 mVpp/Oe, respectively. Such a high slope of $∆V_{3}/∆H_{DC}$ demonstrates that the proposed sensor is quite sensitive to external DC magnetic fields. However, positive and negative DC magnetic fields demonstrate quite a difference in $∆V_{3}/∆H_{DC}$; this may be attributed to the extent to which positive and negative DC magnetic fields change the equivalent elastic modulus $\bar{E}$. Nevertheless, the proposed sensor can still be used to detect DC magnetic fields, as well as to indicate the direction of the DC magnetic field, i.e., positive or negative DC magnetic fields. As can also be seen, the LOD of the DC magnetic field is found to be 0.08 Oe, which is superior to the sensitivities of Hall sensors or magneto-diodes [24,38,56].

### 3.4. Calibration Method for AC and DC Magnetic Field Detection

**Figure 6:**
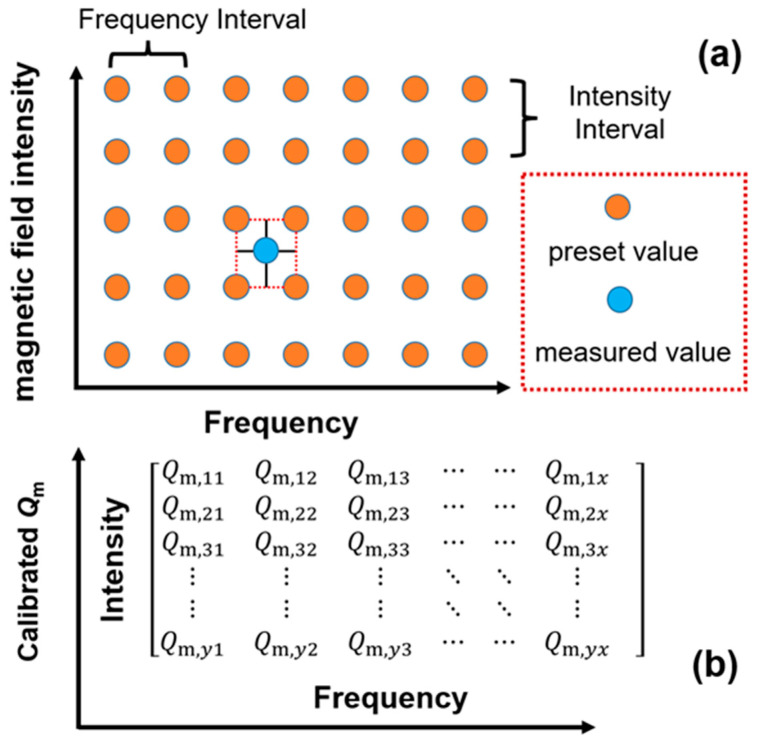
(**a**) The principle of the calibration method for AC magnetic field detection. (**b**) The two-dimensional array of *Q_m_*.

The proposed sensor is experimentally revealed to be effective in both AC and DC magnetic field detection. As for AC magnetic field detection, the output voltage of the proposed sensor has strong dependences on both frequency and intensity. Thus, the output voltage should be calibrated to make it correct for the frequency and intensity measurement. As mentioned before, the frequency dependence can be eliminated by the introducing of a *Q_m_* array. To accurately indicate the frequency as well as intensity of the AC magnetic field, we further expand the *Q_m_* array into a two-dimensional form, which can be used to simultaneously calibrate the frequency and intensity of the AC magnetic field. As shown in Figure 6a, the proposed calibration method is similar to the look-up table method. The table contains preset values, which are arranged in a matrix by frequency and intensity. In each row, the preset values are a group of equidistant nodes that have the same frequency interval. In each column, the preset values are a group of equidistant nodes that have the same intensity interval. As for an AC magnetic field within the testing range, this can be searchable by the look-up table method. The measured value of an AC magnetic field is calibrated by Equations (9) and (10). The preset values are discrete values with both intensity intervals and frequency intervals. In our calibration method, the frequency and intensity intervals are determined to be 2 Hz and 1 Oe, according to the actual test conditions. Meanwhile, the significant digits of the preset values and measured values are different, i.e., the measured value is impossible to be selfsame with the preset value. Thus, the measured value is surrounded by four nodes of the preset values, as shown in Figure 6a.

As for an AC magnetic field, we can search its calibrated value in the array of preset values. The four nodes of preset values contain four pieces of information, including $f_{1}$ and $f_{2}$ for the frequencies and $H_{AC,1}$ and $H_{AC,2}$ for the intensities. Then, the measured values of the AC magnetic field are approximatively determined to be ${(H}_{AC,1}+H_{AC,2})/2$ and ${(f}_{1}+f_{2})/2$, where ${(H}_{AC,1}+H_{AC,2})/2$ is the calibrated intensity and ${(f}_{1}+f_{2})/2$ is the calibrated frequency.

A two-dimensional array of $Q_{m}$ is presented in Figure 6b, in which the number of elements is y×x, where *y* is the number of rows and *x* is the number of columns. And each element in the $Q_{m}$ array, i.e., $Q_{m,yx}$, is acquired by the numerical iterative of Equation (9), based on the curves of ME voltage versus frequency. It is deduced that the precision of the measured value can be greatly improved by increasing the number of preset values. In other words, the more curves of ME voltage versus frequency are transformed into preset values, the higher the testing accuracy. The calibration process can be carried out by a data processing program, where the $Q_{m,yx}$-related preset values are built, together with the integrated computing methods. Based on the proposed calibration method, an arbitrary AC magnetic field within the testing range can be measured with an acceptable accuracy.

When it comes to the situation of DC magnetic field detection, the AC bias magnetic field must be applied to the proposed sensor. Thus, in DC magnetic field detection, the sensor is wrapped with an AC coil. And a signal generation module, as well as a power amplifier module are used to produce the AC bias magnetic field, with the AC frequency around the first-bending mode. According to the experimental results in Figure 5b, the intensity of the DC magnetic field can be quantitatively determined by the fluctuation value of the ME voltage. To calculate the intensity of the DC magnetic field, we make some necessary definitions for the tested results. The output voltage under a fixed AC bias magnetic field is defined as $V_{3,AC}$. The ME voltages under positive and negative DC magnetic fields are, respectively, defined as $V_{3,DC+}$ and $V_{3,DC-}$. The slopes of fluctuation in $V_{3,AC}$ versus positive and negative DC magnetic fields are, respectively, denoted as $\alpha_{ME,DC+}$ and $\alpha_{ME,DC-}$, which are calculated by the ME voltage variation versus the DC magnetic field, as shown in Figure 5b. Then, the measured DC magnetic field can be calculated by the following equations:

(12) $$H_{DC+}=\frac{V_{3,DC+}-V_{3,AC}}{\alpha_{ME,DC+}},$$

(13) $$H_{DC-}=\frac{V_{3,AC}-V_{3,DC-}}{\alpha_{ME,DC-}},$$

where $H_{DC+}$ and $H_{DC-}$ are the positive and negative DC magnetic fields, respectively.

### 3.5. Prototype of the Portable Gaussmeter

**Figure 7:**
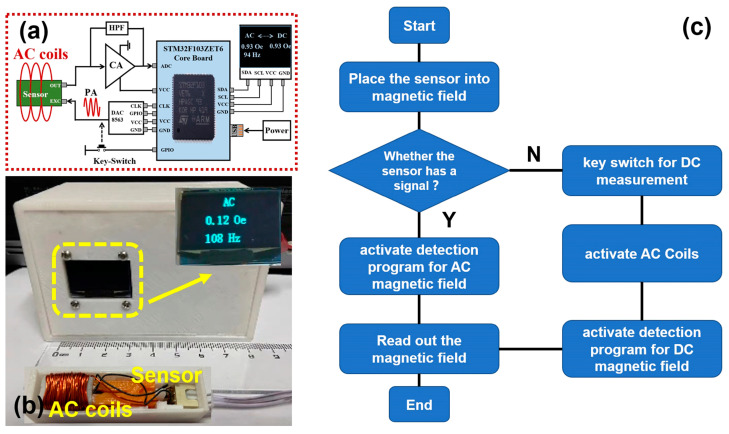
(**a**) The schematic diagram of the component modules for the designed gaussmeter. (**b**) Photograph of the designed gaussmeter. (**c**) The flow chart for AC and DC magnetic field detection.

Finally, the proposed sensor is fabricated into a portable gaussmeter. Figure 7a presents the schematic diagram of the component modules for the designed gaussmeter. The designed gaussmeter consists of such modules as a charge amplification (CA) module (PVA103, Shenzhen Vkinging Electronics Co., Ltd., Shenzhen, China), a high-pass filtering (HPF) module, a Digital-to-Analog Converter module (DAC8563, Texas Instruments Co., Ltd., Dallas, TX, USA), a power amplifier (PA) module, a power module, a single chip microcomputer module, an LCD display module, etc. The power module is powered by a power adapter (or a battery) of 5V 2A. As mentioned above, the detection of the AC and DC magnetic fields can be built into a data processing program. The data processing program is burned into the MCU (Microprogrammed Control Unit, model number: STM32F103ZET6) on the single chip microcomputer. The program burning process is accomplished by the Keil MDK-ARM software (Advanced RISC Machines, Cambridge, UK).

The photograph of the proposed gaussmeter is shown in Figure 7b, where the sensor and modules are packaged into the outer shell made of polylactic acid plastic via 3D printing. The sensor is wrapped by the AC coils, which are powered by the power amplifier (PA) module to supply an AC bias magnetic field for DC detection. The flow chart for both AC and DC magnetic field detection is revealed in Figure 7c. When the proposed sensor is used to detect external magnetic fields, a primary judgment is made regarding whether the sensor already has an output signal. The sensor directly reveals the output signal under the AC magnetic field, while no output signal is detected under the DC magnetic field. If the sensor already has an output signal, it can be deduced that the external magnetic field is the AC type. Then, the detection program for the AC magnetic field is automatically activated. Otherwise, the key switch must be clicked to activate the AC coils and DC magnetic field detection program. The measured result of the external magnetic field is then indicated on the LCD screen. As can be seen in the inset of Figure 7b, the LCD screen indicates the testing result for an AC magnetic field of 0.12 Oe and 108 Hz. The frequency error for AC magnetic field detection is around 1 Hz, as shown in the Supplemental Material video. Being powered by a power adapter (or a battery) of 5V 2A, the proposed gaussmeter has proven to be effective for both DC and AC magnetic field detections, demonstrating its potential for magnetic field detection in distributed application environments. It should be noted that when detecting DC and AC magnetic fields, the proposed sensor operates in different modes. As for AC magnetic field detection, the proposed sensor operates in passive mode, i.e., no working voltage or working current is applied to the sensor. For DC magnetic field detection, an AC exciting coil must be used to provide AC excitation, and the proposed sensor operates in active mode. The proposed sensor is designed to individually detect DC and AC magnetic fields under different modes, not simultaneously detecting DC and AC magnetic fields. As for the environment with both DC and AC superimposed magnetic fields, the sensor should be promoted in its structural design, such as by adopting differential configuration or a Wheatstone bridge structure, so as to decouple the mixed signal of DC and AC superimposed magnetic fields. As also shown in Table 2, the performance comparisons for AC magnetic field detection are made between the proposed sensor and commercial sensors, as well as those sensors reported in other studies. It can be seen that the proposed sensor is competitive among these commercial sensors, such as the Hall sensor, magnetodiode sensor, AMR sensor, etc. Nevertheless, compared with other reported ME sensors, the performance of the proposed sensor still needs to be improved. Further optimizations, such as material component, structure design, preparation technology, etc., could be adopted so as to boost the performance.

## 4. Conclusions

In general, a portable magnetoelectric gaussmeter based on torque effect is proposed in this work. The proposed gaussmeter consists of a cantilever-structured magnetoelectric composite and various modules, including a charge amplification (CA) module, a high-pass filtering (HPF) module, a Digital-to-Analog Converter (DAC) module, a power amplifier (PA) module, a power module, a single chip microcomputer module, an LCD display module, etc. The proposed gaussmeter reveals such performance indexes as an intensity range of 0~10 Oe, frequency range of DC~500 Hz, AC detection limit of 0.01 Oe, DC detection limit of 0.08 Oe, and frequency resolution of 1 Hz. With the assistant of data processing programs, the proposed gaussmeter realizes such functions as data reading, data analyzing, data processing, data displaying, etc. It is revealed that the gaussmeter is portable and effective for both DC and AC magnetic field detections, demonstrating great potential for distributed magnetic field detections, such as electric current examination, magnetic field calibration, etc.

## Figures and Tables

**Table 1:** Material parameter used for finite element simulation.

PZT-5H ceramic	Density $(kg/m^{3}$)	Relative Permittivity	Compliance Coefficient Matrix $(\times 10^{-12}\;1/Pa$)	Piezoelectric Coefficients Matrix $(10^{-10}\;C/N$)
	7500	3400	$\left[\begin{array}{cccccc} 16.5 & -4.78 & -8.45 & 0 & 0 & 0\\ -4.78 & 16.5 & -8.45 & 0 & 0 & 0\\ -8.45 & -8.45 & 20.7 & 0 & 0 & 0\\ 0 & 0 & 0 & 43.5 & 0 & 0\\ 0 & 0 & 0 & 0 & 43.5 & 0\\ 0 & 0 & 0 & 0 & 0 & 42.6 \end{array}\right]$	$\left[\begin{array}{cccccc} 0 & 0 & 0 & 0 & 7.41 & 0\\ 0 & 0 & 0 & 7.41 & 0 & 0\\ -2.74 & -2.74 & 5.93 & 0 & 0 & 0 \end{array}\right]$
Copper	Density $(kg/m^{3}$)	Young modulus (GPa)	Poisson’s ratio	-
	8900	119	0.37	-
NdFeB permanent magnet	Density $(kg/m^{3}$)	Relative permittivity	Young modulus (GPa)	Poisson’s ratio
	7500	1	160	0.24
	Conductivity (S/m)	Relative permeability	Residual flux density (T)	-
	$7\times 10^{5}$	4000	1.01	-

**Table 2:** Performance comparison between the proposed sensor and commercial sensors, as well as those sensors reported in other studies.

Sensors	Sensitivity (mV/Oe)	Resolution (nT)	Size	Reference
Hall sensor (HMIRS GM08)	0.5	2000	-	[24]
Magnetodiode	0.0498–0.0544	-	-	[56]
AMR sensor	3.6	1000	40 × 70 × 2 mm^3^	[57]
GMR sensor	17.5	-	6 × 8 × 8 mm^3^	[58]
TMR sensor	790	0.015 at 1 Hz	12 × 12 mm^2^	[59]
Fluxgate sensor	12,000	0.025 at 1 Hz	35 × 24 × 24 mm^3^	[60]
GMI sensor	390,000	0.38 at 1 Hz	-	[61]
Optical atomic magnetometer	-	10	1300 mm^3^	[62]
Optically pumped magnetometer	-	0.00003	-	[63]
SQUID	-	0.00001	-	[64]
ME sensor	500	0.2 at 1 Hz	25 × 2 × 0.5 mm^3^	[65]
ME sensor	7500	0.025 at 1 Hz	40 × 10 × 0.6 mm^3^	[66]
ME sensor	140,000	0.000135 at 23.23 kHz	100 × 1.5 × 0.6 mm^3^	[30]
ME sensor	81,340	0.00001 at 44.272 kHz	60 × 5 × 0.7 mm^3^	[67]
ME sensor	3.13	10 at 10 Hz	18 × 8 × 6 mm^3^	This work

## Data Availability

The raw data supporting the conclusions of this article will be made available by the authors upon request.

## Supplementary Materials

The following are available online at https://www.mdpi.com/article/10.3390/s25030855/s1, Video S1: Supplemental material-Video.
